# Fecal microbiota in patients with a stoma decreases anaerobic bacteria and alters taxonomic and functional diversities

**DOI:** 10.3389/fcimb.2022.925444

**Published:** 2022-09-14

**Authors:** Shunsuke A. Sakai, Masato Aoshima, Kentaro Sawada, Satoshi Horasawa, Ayumu Yoshikawa, Takao Fujisawa, Shigenori Kadowaki, Tadamichi Denda, Nobuhisa Matsuhashi, Hisateru Yasui, Masahiro Goto, Kentaro Yamazaki, Yoshito Komatsu, Ryota Nakanishi, Yoshiaki Nakamura, Hideaki Bando, Yamato Hamaya, Shun-Ichiro Kageyama, Takayuki Yoshino, Katsuya Tsuchihara, Riu Yamashita

**Affiliations:** ^1^ Graduate School of Frontier Science, Department of Integrated Biosciences, University of Tokyo, Kashiwa, Japan; ^2^ Division of Translational Informatics, Exploratory Oncology Research and Clinical Trial Center, National Cancer Center, Kashiwa, Japan; ^3^ Department of Medical Oncology, Kushiro Rosai Hospital, Kushiro, Japan; ^4^ Translational Research Support Section, National Cancer Center Hospital East, National Cancer Center Hospital East, Kashiwa, Japan; ^5^ Department of Gastroenterology and Gastrointestinal Oncology, National Cancer Center Hospital East, Kashiwa, Japan; ^6^ Department Head and Neck Medical Oncology, National Cancer Center Hospital East, Kashiwa, Japan; ^7^ Department of Clinical Oncology, Aichi Cancer Center Hospital, Nagoya, Japan; ^8^ Divisioin of Gastroenterology, Chiba Cancer Center, Chiba, Japan; ^9^ Department of Gastroenterological surgery Pediatric surgery, Gifu University Hospital, Gifu, Japan; ^10^ Department of Medical Oncology, Kobe City Medical Center General Hospital, Kobe, Japan; ^11^ Cancer Chemotherapy Center, Osaka Medical and Pharmaceutical University Hospital, Takatsuki, Japan; ^12^ Division of Gastrointestinal Oncology, Shizuoka Cancer Center, Shizuoka, Japan; ^13^ Department of Cancer Center, Hokkaido University Hospital, Hokkaido, Japan; ^14^ Department of Surgery and Science, Graduate School of Medical Sciences, Kyushu University, Fukuoka, Japan; ^15^ Department of Radiation Oncology and Particle Therapy, National Cancer Center Hospital East, Kashiwa, Japan; ^16^ Department of Computational Biology and Medical Sciences, Graduate School of Frontier Science, University of Tokyo, Kashiwa, Japan

**Keywords:** gut microbiota, anaerobe, stoma, colostomy, colorectal cancer, 16S rRNA gene

## Abstract

Colorectal cancer (CRC) is one of the most common malignant diseases. Generally, stoma construction is performed following surgery for the resection of the primary tumor in patients with CRC. The association of CRC with the gut microbiota has been widely reported, and the gut microbiota is known to play an important role in the carcinogenesis, progression, and treatment of CRC. In this study, we compared the microbiota of patients with CRC between with and without a stoma using fecal metagenomic sequencing data from SCRUM-Japan MONSTAR-SCREEN, a joint industry-academia cancer research project in Japan. We found that the composition of anaerobes was reduced in patients with a stoma. In particular, the abundance of *Alistipes*, *Akkermansia*, *Intestinimonas*, and methane-producing archaea decreased. We also compared gene function (e.g., KEGG Orthology and KEGG pathway) and found that gene function for methane and short-chain fatty acids (SCFAs) production was underrepresented in patients with a stoma. Furthermore, a stoma decreased Shannon diversity based on taxonomic composition but increased that of the KEGG pathway. These results suggest that the feces of patients with a stoma have a reduced abundance of favorable microbes for cancer immunotherapy. In conclusion, we showed that a stoma alters the taxonomic and functional profiles in feces and may be a confounding factor in fecal microbiota analysis.

## 1 Introduction

Recently, the relationship between the gut microbiome and cancer has been extensively studied (Ternes et al., 2020). Favorable gut microbiotas have been found to be associated with the efficacy of cancer treatment, and their therapeutic applications are also being developed. For example, several commensal bacteria (e.g., *Ruminococcaceae* family, *Akkermansia muciniphila*, *Bifidobacterium longum, Collinsella aerofaciens*, and *Enterococcus faecium*) and taxonomic diversity have been found to correlate with therapeutic efficacy with immune checkpoint inhibitors (ICI) against melanoma, renal cell carcinoma, and non-small cell lung cancer (Gopalakrishnan et al., 2018; Matson et al., 2018; Routy et al., 2018). In addition, studies on fecal microbiota transplantation from responders to ICI therapy to non-responders showed a significant response (Baruch et al., 2021; Davar et al., 2021).

Colorectal cancer (CRC) accounted for 10% of all cancers worldwide in 2020, and its incidence is expected to increase (Xi and Xu, 2021). In patients with CRC, several pathogenic bacteria, such as *Fusobacterium nucleatum* and *Peptostreptococcus anaerobius*, promote carcinogenesis by physically interacting with the tumor (Long et al., 2019; Ternes et al., 2020; Inamori et al., 2021). *Bacteroides fragilis* and *Escherichia coli* are involved in the carcinogenesis and progression of CRC, respectively, by releasing toxins (Haghi et al., 2019; Iyadorai et al., 2020). In contrast, several microbes (e.g., *Lactobacilli* and *Bifidobacteria*) suppress tumor progression as observed in a study using an animal model of CRC (Kim and Lee, 2022). In terms of treatment, *F. nucleatum* in the intestines of patients with CRC has been reported to inhibit the efficacy of 5-fluorouracil and oxaliplatin (Yu et al., 2017). CpG-oligonucleotide immunotherapy has also been used for several cancer types (Chuang et al., 2020). For example, CpG-oligonucleotide immunotherapy in a murine colon carcinoma model was associated with specific gut microbiota, such as *Alistipes* (Iida et al., 2013).

Stoma construction is a surgical procedure performed in patients with CRC and other cases, and it is performed for resection of the primary tumor and palliative stoma as a solution to gastrointestinal obstruction (Amersi et al., 2004; Pickard et al., 2018). In a study examining the quality of life of patients with CRC, 35% of patients with CRC underwent stoma construction (Verweij et al., 2016; Verweij et al., 2017). The number of patients with a stoma is estimated to be approximately 3 million worldwide, and stoma cases are increasing annually (Fortune Business Insights, 2020). Stoma construction opens the intestinal tract and prevents feces from entering the gastrointestinal tract downstream from the stoma. According to a study that examined the relationship between colonic transit time and fecal microbiota, microbial alpha diversity was higher when the fecal transit time through the descending colon was longer (Müller et al., 2020). A study also reported that the composition of the mucosal surface microbiota of the cecum, transverse colon, and sigmoid colon differs from each other (James et al., 2020). Therefore, the unavailability of the colorectal region downstream from stoma because of stoma may affect the analysis of the fecal microbiota. However, to the best of our knowledge, no study has reported the effect of stoma on the fecal microbiota.

In this study, we aimed to analyze the effect of a stoma on the fecal microbiota of patients with CRC. Here, we used 16S rRNA gene sequencing data and clinical data from MONSTAR-SCREEN, industry-academia collaborative cancer research project, to evaluate changes in fecal microbiota between patients with and without stoma. We compared various aspects of the microbiota, including taxonomy, bacteriological characteristics, gene functions, and diversity. Our study revealed the characteristic fecal microbiota of patients with a stoma, and it was found that the stoma can significantly affect the results of fecal microbiota analysis.

## 2 Materials and methods

### 2.1 Study population and samples

SCRUM-Japan MONSTAR-SCREEN is a nationwide tissue and plasma genomic and fecal microbiome profiling study in Japan based on the SCRUM-Japan platform, in which 31 institutions participated. The key inclusion criteria were as follows: i) histopathologically confirmed unresectable or metastatic solid tumors, ii) age ≥20 years, and iii) life expectancy of at least 12 weeks (Nakamura et al., 2021). Fecal samples were collected from eligible patients between December 2019 and June 2021, and written informed consent was obtained. We used a commercial fecal sampling kit with a preservation solution (TechnoSuruga Laboratory Co., Ltd., Shizuoka, Japan). The samples were stored at room temperature for up to seven days and then frozen at -80°C until sample processing. In addition, information on patients’ antibiotic use, proton-pump inhibitor (PPI) use, and the Bristol Scale was collected using a questionnaire.

This study was conducted in accordance with the Declaration of Helsinki and Japanese Ethical Guidelines for Medical and Health Research Involving Human Subjects. The study protocol was approved by the institutional review board of each participating institution and was registered at the University Hospital Medical Information Network (UMIN000036749). This study was conducted in July 2019.

### 2.2 Fecal microbiome analysis

#### 2.2.1 DNA extraction and 16S ribosomal DNA sequencing

A stool suspension (150 μL) in a preservative solution was used for DNA extraction using a NucleoSpin 96 Soil (Macherey-Nagel GmbH & Co. KG, Düren, Germany) according to the manufacturer’s instructions. The final volume of the extraction solution was 100 μL, and approximately 50 μL was obtained. Subsequently, the extracted DNA was purified using Agencourt AMPure XP (Beckman Coulter, Inc., Miami, FL, USA), and the concentration was quantified using the Picogreen dsDNA Assay Kit (Thermo Fisher Scientific, Inc., Waltham, MA, USA).

We used 1 ng of purified DNA (maximum amount for low-concentration samples) as a template for the first PCR using the 16S (V3-V4) Metagenomic Library Construction Kit for NGS (TaKaRa Bio, Inc., Shiga, Japan). PCR products were purified using Agencourt AMPure XP beads (Beckman Coulter, Inc.). The purified PCR product was used as a template for index PCR using a Nextera XT Index Kit (Illumina Inc., San Diego, CA, USA). The indexed PCR products were purified using Agencourt AMPure XP beads (Beckman Coulter, Inc.) and quantified using a Quant-it Picogreen dsDNA Assay Kit (Thermo Fisher Scientific, Inc.). Equimolar mixing of indexed PCR products was performed based on the concentration of each sample to obtain a library. The library size and concentration were calculated using a TapeStation (Agilent Technologies, Inc., Santa Clara, CA, USA). The library was sequenced with the MiSeq sequencer in a multiplex manner, using a 250 bp paired-end sequencing protocol with the MiSeq sequencing reagent kits v3 (Illumina Inc.) and approximately 40–50% of the PhiX Control (Illumina, Inc.).

#### 2.2.2 16S rRNA gene sequencing processing

We employed QIIME2 (v2021.4), a microflora analysis pipeline (Bolyen et al., 2019), for FASTQ annotation. First, low-quality sequences in the FASTQ data were filtered out, and then the DADA2 algorithm was used to correct the error sequences, followed by the generation of a read count table of amplicon sequence variants (ASVs). The reads were rarefied to 42292 reads, which was the minimum number of reads in all samples. Then, a phylogenetic assignment was conducted using the SILVA database (v138) to obtain the number of reads by taxonomy. Next, the unweighted and weighted UniFrac distance matrices were obtained using the QIIME2 command. Finally, principal coordinate analysis (PCoA) was performed on these matrices. All of data are available in **Supplementary Material 1**.

#### 2.2.3 Functional prediction and bacteriological characteristic annotation

We applied PICRUSt2 (v2.4.1) to predict taxonomic function at the gene level in ASV (Douglas et al., 2020). The relative abundance of gene families (KEGG Orthology [KO]) and metabolic pathways (KEGG pathway [pathway]) was determined. Predicted KO copy numbers per ASV were obtained from the PICRUSt2 output files. Subsequently, KOs and ASVs were averaged at the pathway and genus levels, respectively, to calculate the correlation between genus and pathway. The Genomes OnLine Database (GOLD) database (v8) was used to annotate bacteriological features regarding gram-stainability and oxygen requirements (Mukherjee et al., 2021). The microbial metadata in the database were added to the taxonomy in the order of species, genus, order, class, and phylum. The annotation was divided into ‘Gram+’ and ‘Gram-’ for Gram-stainability, and it was also divided into ‘Anaerobe’ and ‘Non-anaerobe’ based on oxygen requirement. If no single annotation could be determined even after referring to the species level, the annotation was classified as ‘Various,’ and if it did not exist in the database, it was classified as ‘Unknown.’

#### 2.2.4 Diversity analysis

Seven α-diversity indices from the relative abundance of ASV, KO, and pathway features per sample were calculated. First, the types of ASV, KO, and pathway features as an index based on richness were obtained, and we defined them as ASV observed, KO observed, and pathway observed, respectively. Shannon indices (ASV Shannon, KO Shannon, and Pathway Shannon) as the diversity of evenness and richness were calculated as follows:


$$H=-\sum_{i=1}^{S}\left(p_{i}log_{2}p_{i}\right)$$

where *p_i_* is defined as each feature ratio, and *S* is the number of features of the ASV, KO, and pathway. Finally, ASV Faith’s PD, an index of the sum of phylogenetic distances between ASVs, was calculated. ASV observed, ASV Shannon, and ASV Faith’s PD were defined as taxonomic diversity, and KO observed, KO Shannon, Pathway observed, and Pathway Shannon were defined as functional diversity.

### 2.3 Statistical analysis

In this study, the relative abundance of features (taxonomy, KO, and pathway), diversity (alpha and beta), and the ratio of taxonomic characteristics (gram stainability and oxygen requirement) were compared between patients with and without a stoma. We detected overrepresented KEGG pathway using Gene Set Enrichment Analysis (GSEA). The ALDEx2 algorithm (v1.24.0) was used to compare the taxonomy, KO, and pathway to determine significant features (Wilcoxon rank-sum test; Benjamini-Hochberg [BH] adjusted *p*-value< 0.3) (Fernandes et al., 2013; Fernandes et al., 2014). The DACOMP (v1.26) algorithm was also used to compare the ALDEx2 results (Brill et al., 2019). To compare the alpha diversity index, the Mann–Whitney U test was used to compare the two groups, and Cohen’s *d* was used as the effect size. A permutation multivariate analysis of variance (PERMANOVA) was performed to test for differences in beta diversity. In a comparison of the gram stainability and oxygen requirement of the microflora, the compositional data were mapped onto the real number space by a centered log-ratio (clr) transformation of each bacteriological characteristic category (e.g., Gram+, Gram-, Anaerobe, and Non-anaerobe) ratio, followed by the Mann–Whitney U test between patients with and without a stoma. For the GSEA method, the effect sizes of KOs that were output by ALDEx2 were arranged in descending order, and ESs were calculated using the GESApy package (v0.10.5). In this case, the threshold for significant enrichment was set at a BH-adjusted *p*-value of< 0.05.

### 2.4 Visualization

Hierarchical fluctuation of taxonomy up to the family level was depicted using the R package Metacoder (v0.3.5) (Foster et al., 2017). Furthermore, a comprehensive graphic display of the KEGG pathway was generated using iPath (v3). The R package of pathview (v1.32.0) was used to illustrate the changes in KOs included in the KEGG pathway (Luo and Brouwer, 2013).

## 3 Results

### 3.1 Study population

**Figure 1:**
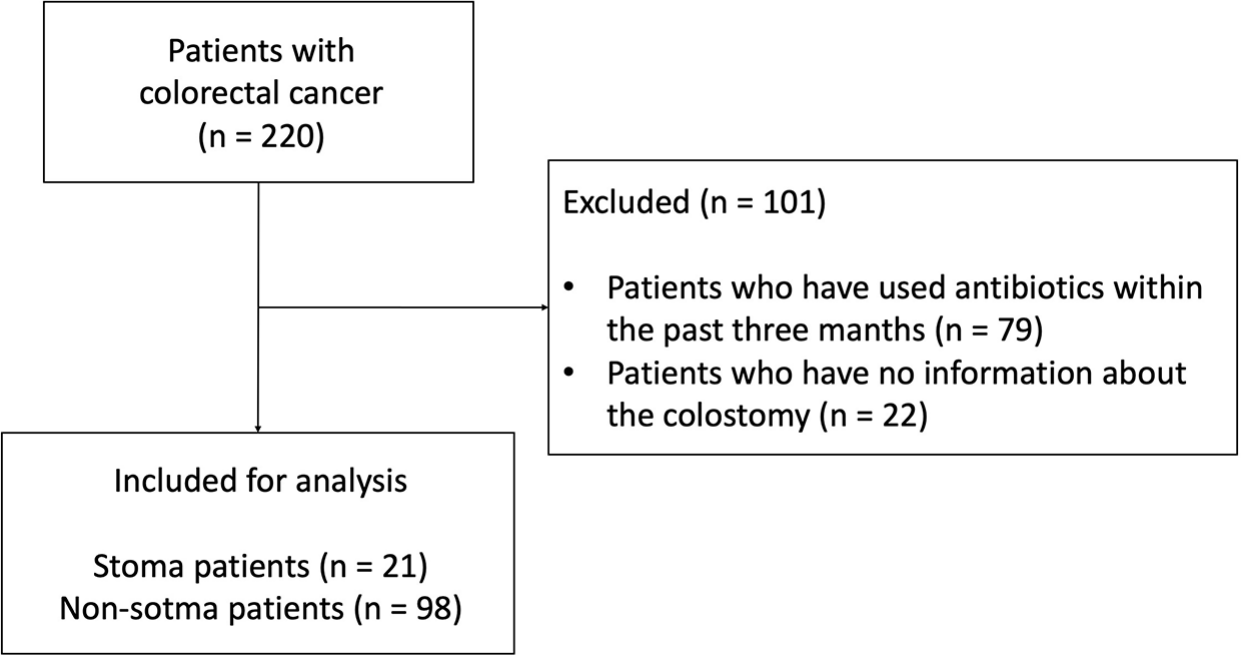
Consort diagram of this study.

The 16S rRNA gene sequence data of fecal samples from 220 patients with CRC before chemotherapy enrolled in MONSTAR-SCREEN were available. Of these 220 patients, we excluded 79 antibiotic users based on a questionnaire and 22 patients without information on stoma. Finally, pre-treatment fecal sample data from 119 patients (21 patients with a stoma [stoma group] and 98 patients without a stoma [non-stoma group]) were used in this study (**Figure 1**). In the stoma group, stomas were present in the small intestine, transverse colon, descending colon, and sigmoid colon (**Supplementary Table 1**). All patients in the analysis had stage IV CRC and an ECOG-PS<2. The clinical features of the patients are shown in **Table 1**. The age and BMI (mean ± [SD]) in the stoma group were 58 ( ± 17.2) and 20.9 ( ± 3.9), respectively. We also compared the frequency of consumption of fermented foods and drinking (**Supplementary Table 2**). We used the Bristol scale to evaluate fecal consistency, and the results showed that patients in the stoma group had soft stools (chi-square test: *p*< 0.001) (**Supplementary Table 3**). There were no other significant differences in any of the characteristics (e.g., gender, proton pump inhibitors, primary tumor resection, and primary tumor location).

**Table 1:** Patient characteristics.

	Stoma group	Non-stoma group	
**Characteristics**	**N = 21^1^**	**N = 98^1^**	***p*-value^2^**
**Age (years)**	58 (24–84)	63 (38–85)	0.2
**BMI (kg/m^2^)**	21 (3.9)	23 (3.5)	0.07
Unknown	2	0	
**Gender**			1
Female	9 (43%)	44 (45%)	
Male	12 (57%)	54 (55%)	
**PPI**			0.3
User	16 (76%)	84 (86%)	
Non-user	5 (24%)	14 (14%)	
**Primary tumor resection** Yes	12 (57%)	67 (68%)	0.3
No	9 (43%)	31 (32%)	
**Location of the primary tumor**			0.2
Cecum	0 (0%)	7 (7.3%)	
Ascending colon	0 (0%)	10 (10%)	
Transverse colon	3 (15%)	15 (16%)	
Descending colon	2 (10%)	3 (3.1%)	
Sigmoid colon	5 (25 %)	31 (32%)	
Rectum	10 (50%)	30 (31%)	
No information	1	2	

^1^Mean (range); Mean (SD); n (%)

^2^Welch Two Sample t-test; Fisher's exact test; Pearson's Chi-squared

BMI, body mass index; PPI, proton-pump inhibitor.

### 3.2 Taxonomic composition of each sample and their characteristics

**Figure 2:**
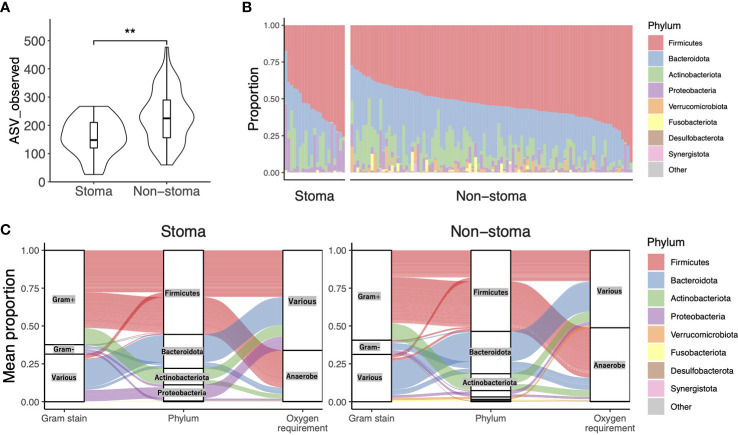
Taxonomic alpha diversity in patients with or without a stoma. **(A)** Violin plot of taxonomic richness (ASV observed). Significance differences between the two groups were tested using the Mann–Whitney U test. **p< 0.01. **(B)** The composition of phylum-level bacteria. **(C)** The Sankey diagram shows the relationship between taxonomic composition at the phylum level, its Gram-stainability, and whether it is anaerobe or not. The vertical axis represents the average composition per stoma group or non-stoma group.

To confirm the change in bacterial composition in feces due to stoma, we obtained the number of ASV, a taxonomic unit classified by a single base change, after correcting for sequence errors. We observed that the number of ASV decreased in the stoma group (*p* = 0.001) (**Figure 2A**). We then analyzed the taxonomic composition and found that Archaea were present specifically in the non-stoma group (*p* = 0.04) (**Supplementary Figure 1A**). At the phylum level, *Firmicutes* (average relative abundance in the stoma group [S] = 56%, non-stoma group [N] = 54%), *Bacteroidota* (S = 22%, N = 28%), *Actinobacteriota* (S = 11%, N = 11%), and *Proteobacteria* (S = 11%, N = 4.2%) were predominant in both groups (**Figure 2B**). Comparing the two groups, *Firmicutes* (BH adjusted *p*-value = 0.04) and *Proteobacteria* (*p* = 0.1) were more abundant in the stoma group, whereas *Verrucomicrobiota* (*p* = 0.1) and *Desulfobacterota* (*p* = 0.06) were less abundant. These results showed that the phylum in feces was different in the stoma group than in patients without a stoma. In addition, focusing on the features of gram-stainability and anaerobicity, we used the Genomes OnLine Database (GOLD) to annotate these bacteriological features and compared their relative abundances between the two groups. There was no significant difference in the frequency of gram-positive (S = 62%, N = 59%; *p* = 0.2) and gram-negative (S = 6.3%, N = 9.4%; *p* = 0.1) bacteria between the two groups. In contrast, there was a significant decrease in the relative abundance of obligate anaerobes (S = 34%, N = 49%; *p*< 0.001) in the stoma group (**Table 2**, **Supplementary Figure 1B**). The Sankey diagram in **Figure 2C** shows the relationship between taxonomic composition at the phylum level, gram-stainability, and anaerobicity. Interestingly, although the results showed no large difference in the relative amounts of *Firmicutes*, which is generally considered anaerobic, the taxonomy annotated as anaerobic was greatly reduced in the stoma group. This result implied that a stoma was responsible for reducing obligate anaerobes in stools.

**Table 2:** Comparison of Gram-stainability and oxygen requirement with and without a stoma.

Categories		Cohen's *d* ^1^	*p*-value^2^	*fdr* ^3^
**Gram-stainability**	Gram+	0.23	0.2	0.2
	Gram-	-0.29	0.1	0.2
	Various	0.066	0.7	0.7
	Unknown	0.15	0.1	0.2
**Oxygen requirement**	Anaerobe	-0.86	**< 0.001**	**0.003**
	Non-anaerobe	0.51	**0.02**	**0.04**
	Various	-0.11	0.5	0.5
	Unknown	0.30	0.5	0.5

^1^Effect size (stoma group – non-stoma group); Criteria: d = 0.2 (small), d = 0.5 (medium), d = 0.8 (large)

^2^Mann–Whitney U-test

^3^p-value corrected using the Benjamini-Hochberg method

The p-value shown in bold indicate significant differences (p<0.05).

### 3.3 Stoma-induced variation in diversity and taxonomy

**Figure 3:**
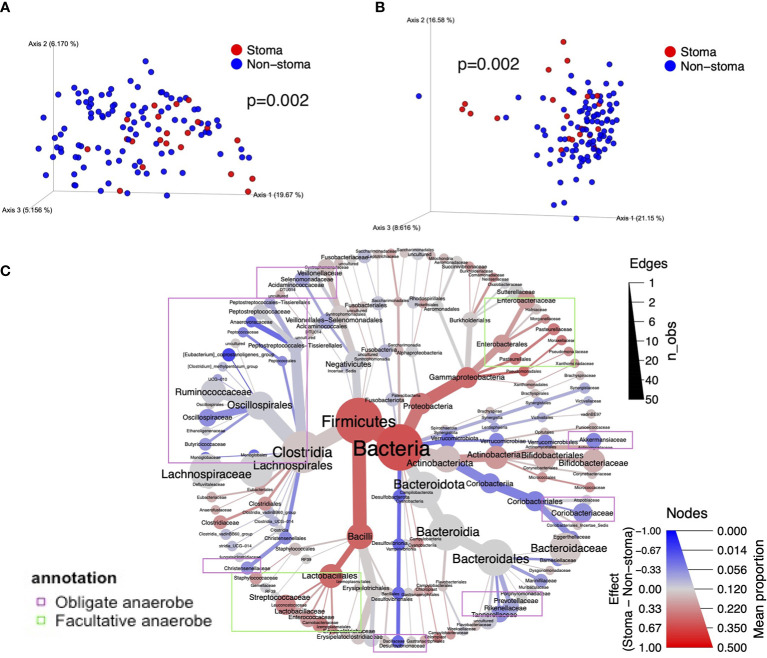
The difference in taxonomic beta diversity between the stoma group and non-stoma group. **(A)** PCoA plot of unweighted UniFrac distance. **(B)** PCoA plot of weighted UniFrac distance. **(C)** Heat tree with the effect size illustrates the hierarchical differences of bacteria. The color represents the effect size of the comparison in ALDEx2. The size of the nodes represents the average percentage of taxonomy, and the size of the edges represents the number of observations of genus-level bacteria belonging to the taxon of the parent node. Taxa surrounded by purple borders include obligate anaerobes, while taxa surrounded by light green borders include facultative anaerobes and aerobes. PCoA; Principal Coordinate Analysis.

Next, we investigated the difference in beta-diversity between the stoma and non-stoma groups *via* principal coordinate analysis (PCoA) using unweighted and weighted UniFrac distances (**Figure 3A, B**). These results showed significant taxonomic discrimination between the two groups. We subsequently performed permutation multivariate analysis of variance (PERMANOVA) with 999 permutations (unweighted UniFrac distance, *F* = 3.3, *p* = 0.002; weighted UniFrac distance, *F* = 4.5, *p* = 0.002). These results also showed significant quantitative and qualitative taxonomic discriminations between the two groups. The ALDEx2 algorithm was then used to examine the differences in the relative abundance of bacteria at each taxonomic level between the two groups, and a phylogenic heat map tree with the effect sizes is shown (**Figure 3C** and **Supplementary Table 4**). We found a tendency to decrease obligate anaerobes such as *Anaerovoracaceae, Oscillospiraceae, Desulfovibrionaceae, Rikenellaceae*, and *Akkermansiaceae* (BH adjusted *p*-value< 0.3, effect size< -0.17), at the family level in the stoma group. In contrast, facultative anaerobes and aerobes such as *Enterococcaceae, Pasteurellaceae, Pseudomonadaceae, Campylobacteraceae*, and *Morganellaceae* increased (BH adjusted *p*-value< 0.3, effect size > 0.17). We also observed that *Clostridiaceae* abundance was increased in the stoma group. At the genus level, *Monoglobus* (BH adjusted *p* = 0.1), *Akkermansia* (*p* = 0.3), *Alistipes* (*p* = 0.1), *Anaerotruncus* (*p* = 0.3), *Fusicatenibacter* (*p* = 0.3), *Intestinimonas* (*p* = 0.2), and *Dorea* (*p* = 0.2) were decreased in the stoma group. Meanwhile, *Acinetobacter* (*p* = 0.2), *Haemophilus* (*p* = 0.2), and *Enterococcus* (*p* = 0.2) increased (**Supplementary Figure 2**). The result was validated by another statistical tool, DACOMP, and we observed that 87% of the genera detected by ALDEx2 were also detected DACOMP (**Supplementary Figure 3**). These results also suggest that obligate anaerobes decreased, and other bacteria that could tolerate the oxygen environment increased in the stoma group. Comparing the number of ASVs between each stoma location (small intestine, right-side colon, and left-side colon) and the non-stoma group confirmed decreased fecal taxonomic diversity in all stoma locations compared with the non-stoma group. The results also showed that the stoma of the small intestine had a lower number of ASVs than that of the right-side colon. (**Supplementary Figure 4A**). *Akkermansia* decreased in all stoma locations compared to that in the non-stoma group, while *Alistipes* decreased in the stoma of the small intestine and right-side colon (**Supplementary Figure 4B**).

### 3.4 Analysis of the difference in gene function inferred from bacteria between patients with and without a stoma

**Figure 4:**
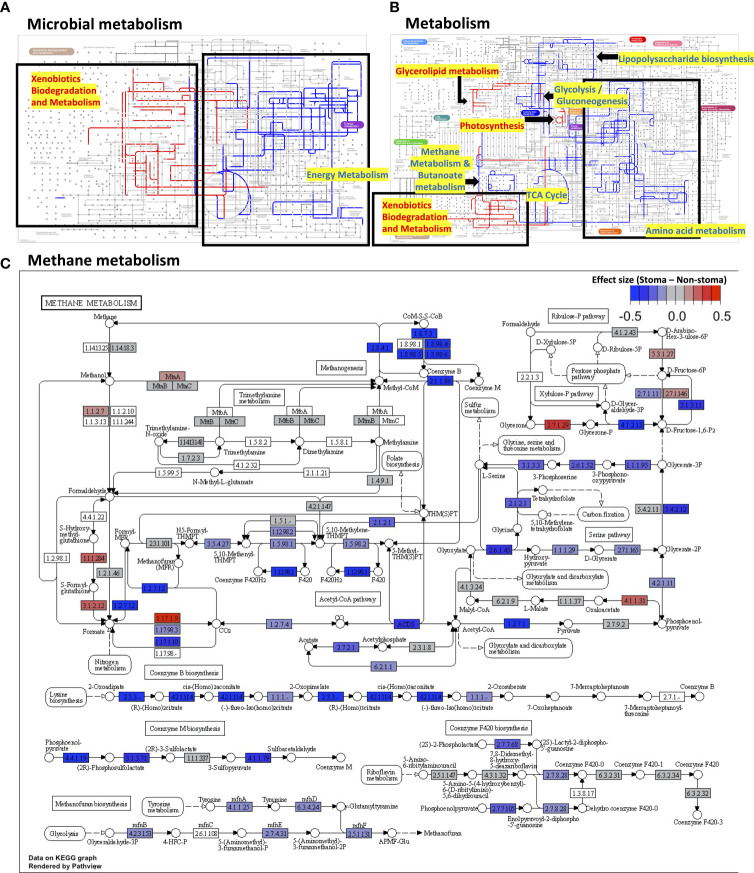
The difference in gene functions between the stoma group and non-stoma group. **(A)** Mapping pathway enrichment analysis results in microbial metabolism using Ipath. The red edges indicate enrichment in patients with a stoma, the blue indicates enrichment in patients without a stoma, the light gray indicates no significant difference, and the gray indicates pathways do not present in both groups. Pathways with no edges indicate that they were not covered by GSEA. **(B)** Overall metabolism pathway. **(C)** Methane metabolism. The color represents the effect size of the comparison (with a stoma vs. without a stoma) in ALDEx2. GSEA; Gene Set Enrichment Analysis.

Next, we focused on the gene functions of patients with a stoma. We used PICRUSt2 to infer the gene functions of the microbiota and annotated KEGG Orthology (KO) against them. We applied the ALDEx2 algorithm to compare the relative abundance of KOs between the stoma and non-stoma groups. There was a significant reduction in the stoma group for glycolate oxidase FAD-binding subunit (K11472; BH adjusted *p-*value = 0.04), dimethyl sulfoxide reductase membrane subunit (K00185; *p* = 0.05), and acetate CoA/acetoacetate CoA-transferase alpha and beta subunits (K01034; *p* = 0.1, K01035; *p* = 0.1) (**Supplementary Table 5**). This result implies that the stoma reduced redox and SCFAs production. To evaluate which gene functions fluctuated at the metabolic pathway level, we subsequently performed Gene Set Enrichment Analysis (GSEA) to calculate the enrichment score (ES) of functional units in the KEGG pathway using all effect sizes of KOs (**Supplementary Table 6**). **Figures 4A, B** show the enriched pathways in the global metabolic maps. xylene degradation, benzoate degradation, glycerolipid metabolism, and photosynthesis were enriched in the stoma group (BH adjusted *p*< 0.05, ES > 0). In contrast, many pathways were decreased in the stoma group, such as methane metabolism, butanoate metabolism, and other amino acid metabolisms (BH adjusted *p*< 0.05, ES< 0). Focusing on the alteration of KOs belonging to methane metabolism, we found a decreasing tendency (BH adjusted *p-*value< 0.3, effect size< -0.17) for KOs such as heterodisulfide reductase subunits C2, B2, and A2 (1.8.7.3, 1.8.98.4, 1.8.98.5, 1.8.98.6), phosphosulfolactate synthase (4.4.1.19), and methanogen homocitrate synthase (2.3.3.-) in the stoma group (**Figure 4C**). These results suggest that a stoma increased the microbial pathway of xenobiotic biodegradation and metabolism but decreased that of anaerobic metabolism.

### 3.5 Investigation of taxonomic and their functional diversity index

**Figure 5:**
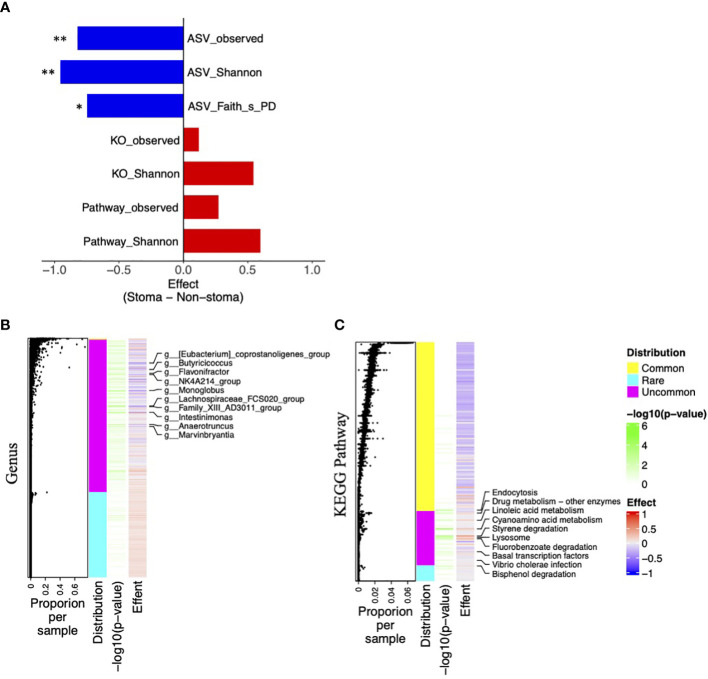
Difference of taxonomic and functional diversity indices between the stoma group and non-stoma group and distribution of features. **(A)** Bar plot of effect size when comparing taxonomic (ASV) and functional (KO and Pathway) diversity indices between two groups. Significance differences between the two groups were tested using the Mann–Whitney U test. * *p*< 0.05, ** *p*< 0.01. **(B)** Heatmap of taxonomic genus-level effect sizes, annotated with jitter plots representing average composition per sample of features. Significance differences between the two groups were tested using Fisher’s exact test. The labels on the vertical axis indicate genera with *p*-values less than 0.05. **(C)** Pathway effect sizes.

To consider the changes in bacteria and their functions, we calculated various diversity indices and compared them between the stoma and the non-stoma groups. ASV, the output of QIIME2, is the unit that divides the taxonomy by a single nucleotide change after correcting for sequencing errors, KO is the gene set at the functional level inferred from the ASV using PICRUSt2, and the KEGG pathway is the sum of KOs belonging to each unit of the KEGG pathway. After obtaining the ASV, KO, KEGG pathway, we calculated Shannon diversity indices of those three categories and Faith’s phylogenetic distance (Faith’s PD) for ASVs. We found that the ASV observed, ASV Shannon, and ASV Faith’s PD were significantly decreased in the stoma group (ASV observed, *p* = 0.001; ASV Shannon, *p* = 0.001; ASV Faith’s PD, *p* = 0.02; Mann–Whitney U-test, BH adjusted *p*-value). In contrast, KO observed, KO Shannon, Pathway observed, and Pathway Shannon indices tended to increase in the stoma group (KO observed, *p* = 0.2; KO Shannon, *p* = 0.1; Pathway observed, *p* = 0.1; Pathway Shannon, *p* = 0.06) (**Figure 5A**). These results indicated that a stoma decreased taxonomic diversity but increased functional diversity.

Next, to elucidate the responsible genera and functions, we used effect size and presence per sample (*p*-value; Fisher’s exact test) in the stoma and non-stoma cases (**Figures 5B, C**). We divided their features into three categories: >99% presence, 1–99% presence, and<1% presence, and defined them as “Common”, “Uncommon”, and “Rare”, respectively. “Uncommon genera” and “Uncommon Pathway” showed significant differences of presence/absence ratio. Importantly, the effect size of “Uncommon genera” and “Uncommon Pathway” tended to coincide with the increase or decrease of the taxonomic and functional diversity in **Figure 5A**, respectively. These results suggested that “Uncommon genera” and “Uncommon Pathway” were responsible for increasing or decreasing diversity.

Notably, we observed contradictory tendencies between taxonomic and functional diversities (KO and pathway). We used PICRUSt2 to predict potential KOs and pathways, and the PICRUSt2 algorithm estimated the gene function of the microbiota by mapping ASVs onto a phylogenetic tree of taxa whose genomes have been sequenced. We examined the predicted KO copy numbers per ASV from the PICRUSt2 output. We identified the genus responsible for pathway richness among the features with a *p*-value of Fisher’s exact test< 0.1. In **Supplementary Figure 5**, when the z-score was higher, there was a strong correlation between microbes and pathways. Most “Uncommon genera” did not influence the “Uncommon Pathway”,and several “Uncommon genera” and “Rare genera” such as *Acinetobacter*, *Pseudomonas*, and *Campylobacter* related to xenobiotic degradation, one of the “Uncommon Pathway”. These results illustrate that the stoma reduced many microbes with common functions but increased a few microbes with unique functions, which exemplified the conflicting changes in taxonomic and functional diversities.

## 4 Discussion

Stoma construction is a medical procedure performed in patients with colon cancer and is widely used worldwide (Amersi et al., 2004). However, there have been no reports analyzing changes in fecal microbiota in the presence or absence of a stoma. In this study, we used 16S rRNA gene sequencing analysis of 119 samples to infer fecal taxonomic composition and function and compared them between patients with and without a stoma.

Our metagenomic analysis revealed that a stoma decreased taxonomic diversity and obligate anaerobes, such as *Akkermansia, Intestinimonas*, and *Alistipes*. In addition, methane-producing archaea, such as *Methanobrevibacter* and *Methanosphaera*, were not detected in the stoma group, and the gene functions related to methane synthesis were consistently reduced in patients with a stoma. The taxonomic diversity (e.g., ASV observed, ASV Shannon, and ASV Faith’s PD) of fecal microbiota increased as it transitioned through the descending colon (Müller et al., 2020). *Alistipes* and *Akkermansia* have been reported to be preferentially present in the intestinal mucosa on the left side rather than on the right side (Flemer et al., 2017). Moreover, methanogens were more prevalent in the left-side colon than in the right-side (Macfarlane et al., 1992). Genes involved in methane production have also been reported to be overrepresented in the left colon (Nava et al., 2012). A stoma creates an opening of the ileum or colon; that is, patients with a stoma reduce fecal transit of the distal colorectum, where diversity of microbiota is high, and certain bacteria are present in higher abundance. Consistent with these findings, our results showed that fecal taxonomic diversity and *Alistipes* tended to decrease when the stoma was located in the proximal gastrointestinal tract compared with that in the distal region. Furthermore, a comparison based on the Bristol scale showed that stool form and hardness were strongly correlated with fecal bacterial richness, *Akkermansia*, and *Methanobrevibacter* (Vandeputte et al., 2016). We showed that most fecal samples from patients with a stoma had soft stools. Therefore, microbiota and taxonomic diversity changes in patients with a stoma may be attributed to low stool hardness and a decreased fecal transition period in the left-side colonic region. Notably, our observations also implied that the overall population of obligate anaerobes was lower in patients with a stoma. A study in mouse models showed that luminal oxygen partial pressure decreased from the proximal to the distal colon (He et al., 1999). As mentioned above, the feces of patients with a stoma transit less to the distal colon region. Altogether, it is suggested that the feces of patients with a stoma do not transit to the colonic region with the lowest partial oxygen pressure in the gut; therefore, we observed a decreasing population of obligate anaerobes. These previous reports and our observations imply that the changes in the microbiota of patients with a stoma are attributed to their intestinal environment rather than that of the stoma pouch.

Interestingly, functional diversity and many gene functions related to xenobiotics, such as styrene, xylene, and benzoate degradation, were enriched in patients with a stoma. We also found an increase in aerobic bacteria such as *Enterococcus*, *Pseudomonas*, and *Acinetobacter* in patients with a stoma. These bacteria are responsible for the gene functions related to xenobiotics (Desouky, 2003; Fernández et al., 2009; Saffarian et al., 2017) and can cause wound and suppurative infections (Bryanti and Hammond, 1974; Mihu et al., 2010; Doughari et al., 2011; Kim et al., 2015). In addition, many patients (21%–70%) with a stoma have complications, including peristomal infection (Shabbir and Britton, 2010). According to a previous study on surgical site infections due to stoma reversal surgery, these patients might have been infected with *Enterococcus* and *Pseudomonas* (Liang et al., 2013). Combining these observations, a high abundance of *Pseudomonas*, *Acinetobacter*, and *Enterococcus* in feces was associated with peristomal infection.

Several reports have shown that fecal taxonomic diversity is positively correlated with the efficacy of immune checkpoint inhibitors (ICI) in patients with non-small cell lung cancer (NSCLC), renal cell carcinoma (RCC), and malignant melanoma (MMe) (Gopalakrishnan et al., 2018; Matson et al., 2018; Routy et al., 2018). At each taxon level, ICI responders with NSCLC and RCC had a higher abundance of *Akkermansia, Intestinimonas*, and *Alistipes* (Routy et al., 2018; Derosa et al., 2022), and the responders with MMe and NSCLC also had many methanogenic archaea and *Methanobrevibacter*, respectively (Simpson et al., 2020; Song et al., 2020). In addition, *Alistipes* positively affects the efficiency of CpG-oligonucleotide immunotherapy in CRC (Iida et al., 2013). Many of these bacteria are short-chain fatty acids (SCFAs) producers (Derrien et al., 2004; Bui et al., 2015; Venegas et al., 2019; Parker et al., 2020). In this study, SCFA-related gene function was also coherently decreased in stoma cases. SCFA-producing bacteria and methanogens are known to be involved in host immunomodulation (Bang et al., 2014; Chaudhary et al., 2018; Venegas et al., 2019), which may be a mechanism that influences tumor invasion immunity in cancer therapy (Nomura et al., 2020; Luu et al., 2021). This indicates that the relative abundance of the so-called favorable bacteria in cancer immunotherapy is reduced in the feces of patients with a stoma.

This study has several limitations. First, there may be a time gap between defecation and stool collection in patients with a stoma, and to our knowledge, no study has evaluated how fecal microbial composition changes when feces are left in a clean and enclosed space such as a stoma pouch. We have already discussed that the changes in the fecal microbiota were caused by a decrease in the fecal transit period in the left-side colonic region, low stool hardness, and peristomal infection. Therefore, these changes may not be due to the exposure time in the environment of the stoma pouch. Furthermore, in this study, the small sample size of patients with a stoma was not sufficient to detect stoma location-specific microbiota characteristics. A more detailed discussion may be possible if a larger sample size of patients with a stoma is available.

In conclusion, we investigated the fecal microbiota of patients with CRC and compared the stoma and non-stoma groups. These results showed a significant difference in the fecal microbiota of patients with stoma compared with those without a stoma, and observed differential microbiota was related to the efficacy of cancer immunotherapy. Thus, we caution against interpreting the results of the gut microbiota analysis in patients with a stoma.

## Data availability statement

The datasets presented in this study can be found in online repositories. The names of the repository/repositories and accession number(s) can be found below: https://qindao.hgc.jp/cgi-bin/files/Sakai_et_al_readcount_table.xlsx.

## Ethics statement

The studies involving human participants were reviewed and approved by University Hospital Medical Information Network (UMIN000036749). The patients/participants provided their written informed consent to participate in this study.

## Author contributions

SS, MA, and RY designed the study, and performed the research, analyzed the data, and contributed equally. SS, MA, RY, YH, SK, KS, SH, AY, TF, YN, KN, HB, TY, and KT provided scientific insight into this study. SS wrote the manuscript. RY and KT supervised the study and revised the manuscript accordingly. All members have read the manuscript and approved the content.

## Funding

This study was supported by the National Cancer Center Research and Development Fund (grant number 31-A-10). This study was also supported by AMED Seeds A (grant number CPOT, 21-A-33).

## Acknowledgments

We thank all members of the SCRUM-Japan and MONSTAR teams. We also thank the lab members.

## Conflict of interest

SK reports honoraria from Eli Lilly, Taiho, Ono, Bristol-Myers Squibb, Chugai, Bayer, Merck Serono, Daiichi Sankyo, Eisai; and research funding from Taiho, Eli Lilly, MSD, Chugai, Nobelpharma, Ono, Daiichi Sankyo, and Yansen.TD reports honoraria from Sawai Pharmaceutical; speakers’ bureau from Sysmex; and research funding from MSD and Ono Pharmaceutical. HY reports honoraria from Taiho Pharmaceutical, Chugai, Bristol-Myers Squibb, Daiichi-Sankyo, Terumo, Eli Lilly Japan, Merck Biopharma, Yakult Honsha, and Bayer Yakuhin; and research funding from MSD, Daiichi-Sankyo, and Ono Pharmaceutical. MG reports honoraria from Yakult Honsha, Taiho Pharmaceutical, Daiichi-Sankyo, Eisai, and Ono Pharmaceutical; and research funding from Chugai, Taiho Pharmaceutical, Nippon Kayaku, Ono Pharmaceutical, and Eli Lilly. YK reports honoraria from Asahi Kasei Pharma, Bayer Yakuhin, Bristol-Myers Squibb., Chugai, Daiichi-Sankyo, Eli Lilly, Kyowa Kirin, Medical Review, Merck Biopharma, Mitsubishi Tanabe Pharma, Moroo, Nipro, Ono Pharmaceutical, Pfizer Japan, Sanofi, Shire Japan, and Taiho Pharmaceutical; and research funding from A2 Healthcare, Asahi Kasei Pharma, Astellas Pharma, Bayer Yakuhin, Daiichi-Sankyo, Sumitomo Dainippon Pharma, Eisai, Mediscience Planning, NanoCarrier, Ono Pharmaceutical, Parexel International, Sanofi-aventis, Shionogi & Co., Ltd., Taiho Pharmaceutical, Yakult Honsha, Incyte, IQVIA, MSD, Nippon Zoki Pharmaceutical, Syneos Health Clinical, and Sysmex. YN reports research grants from Taiho Pharmaceutical, Chugai Pharmaceutical, Guardant Health, Genomedia, Daiichi Sankyo, Seagen, and Roche Diagnostics. HB reports honoraria from Taiho Pharmaceutical, Lilly Japan, Takeda Pharmaceutical, Chugai, Sanofi, and Yakult Honsha: and research funding from AstraZeneca and Sysmex. TY reports honoraria from Taiho Pharmaceutical, Chugai Pharmaceutical, Eli Lilly, Merck Biopharma, Bayer Yakuhin, Ono Pharmaceutical, and MSD; and research funding from Ono Pharmaceutical, Sanofi, Daiichi Sankyo, PAREXEL International, Pfizer Japan, Taiho Pharmaceutical, MSD, Amgen, Genomedia, Sysmex, Chugai Pharmaceutical, and Nippon Boehringer Ingelheim KT reports honoraria from SRL Diagnostics, DNA Chip Research, Chugai, Novartis, Takeda, MSD, Sysmex, Nippon Kayaku, Illumina, Fujitsu, Varian Medical Systems, Miyarisan Pharmaceutical, Bristol-Myers Squibb, AstraZeneca, and Novartis. RY reports honoraria from Takeda Pharmaceutical.

The remaining authors declare that the research was conducted in the absence of any commercial or financial relationships that could be constructed as a potential conflict of interest.

## Publisher’s note

All claims expressed in this article are solely those of the authors and do not necessarily represent those of their affiliated organizations, or those of the publisher, the editors and the reviewers. Any product that may be evaluated in this article, or claim that may be made by its manufacturer, is not guaranteed or endorsed by the publisher.
