# Supplementary material for: Fecal microbiota in patients with a stoma decreases anaerobic bacteria and alters taxonomic and functional diversities
Source: Front Cell Infect Microbiol. 2022 Sep 14;12:925444. doi: 10.3389/fcimb.2022.925444 (PMC9515963; doi:10.3389/fcimb.2022.925444)
Supplement: Supplementary file 1 [file DataSheet_1.docx]

Supplementary Material

# Supplementary Data

## Supplementary Figures

**Supplementary Figure 1.** (A) Archaeal composition at the genus level. (B) Microbial composition in terms of Gram staining and oxygen requirement.

**Supplementary Figure 2.** Comparison of bacteria at the genus level between patients with and without stoma. The bar plot shows the effect size: the output of ALDEx2.

**Supplementary Figure 3.** Concordance of genera with adjusted p-value<0.3 in ALDEx2 or DACOMP.

**Supplementary Figure 3.** (A) Boxplot showing the ASV observed by stoma location. (B) Relative abundances of *Akkermansia* and *Alistipes*. Significant differences are calculated using the Mann–Whitney U test with a BH-adjusted p-value of <0.3.

**Supplementary Figure 4.** Heatmap representing the relationship between pathway and genus, which is annotated with effect size: output of ALDEx2, *p*-value: Fisher’s exact test, and defined distribution of genus and pathway. The genus and pathway features were focused on when the *p*-value of Fisher’s exact test was <0.1. The text annotations on the right indicate genera with strong correlations with the pathways.
